# Identification of a pyroptosis-related long non-coding RNA Signature for prognosis and its related ceRNA regulatory network of ovarian cancer

**DOI:** 10.7150/jca.88485

**Published:** 2023-09-25

**Authors:** Haoya Xu, Miao Lu, Yuna Liu, Fang Ren, Liancheng Zhu

**Affiliations:** 1 Department of Obstetrics and Gynecology, Shengjing Hospital of China Medical University, Shenyang 110004, Liaoning, China.; 2 Obstetrics and Gynecology Hospital, Fudan University, Shanghai 200011, China.; 3 Shanghai Key Laboratory of Female Reproductive Endocrine Related Diseases, Shanghai 200011, China.

**Keywords:** pyroptosis-related genes, lncRNA, ovarian cancer, immune microenvironment, ceRNA regulatory network

## Abstract

**Aim:** To identify the pyroptosis-related long non-coding RNAs (lncRNAs) in ovarian cancer and construct a prognostic signature based on them.

**Methods:** Expression data from TCGA was used to explore differentially expressed pyroptosis-related lncRNAs in ovarian cancer. A risk signature was established by LASSO and cox regression analysis and then validated. Databases such as ESTIMATE, CIBERSORT, TIMER, XCELL were used to identify the relation between this signature and the immune microenvironment of ovarian cancer. Gene Set Enrichment Analysis was introduced to identify the pathways and functions that the signature may participate in. Based on miRcode and starBase databases, microRNAs related to the lncRNAs in our signature and the positively co-expressed pyroptosis- related genes were screened and a competing endogenous RNA (ceRNA) network was then constructed. Quantitative reverse transcription PCR was conducted to validate the expression levels of two lncRNAs in this ceRNA network.

**Results:** A 13 pyroptosis-related lncRNA prognostic signature (MYCNOS, AL161772.1, USP30-AS1, ZNF32-AS2, AC068733.3, AC012236.1, AC015802.5, KIAA1671-AS1, AC013403.2, MIR223HG, KRT7-AS, PTPRD-AS1 and LINC01094) was constructed. Patients in high-risk group had a significantly worse prognosis than that of low-risk (P<0.0001). Immune infiltration analysis found that patients identified as high-risk had a higher infiltration of macrophages and tumor-associated fibroblasts. Further pathway analysis revealed that the signature may be involved in epithelial mesenchymal transition, extracellular matrix receptor interaction, and focal adhesion. Finally, a competitive endogenous inhibition relationship was discovered between LINC01094, KRT7-AS, MYCNOS, ZNF32-AS2, AC012236.1 and pyroptosis- related genes such as *IRF1*, *NOD1*, *GSDMC*, *NLRP1*, *PLCG1*, *GSDME* and *GZMB*, in which LINC01094 and KRT7-AS were found to be overexpressed in three ovarian cancer cell lines.

**Conclusion:** We constructed a pyroptosis-related lncRNA signature and correlate it to the immune microenvironment. A ceRNA regulatory network related to pyroptosis was also constructed, which provides novel insights useful for the study of pyroptosis in ovarian cancer.

## 1. Introduction

Ovarian cancer is the deadliest gynecological malignancy 1, and approximately 230,000 people are diagnosed every year 2. Owing to the lack of observable symptoms in early-stage patients, approximately 75% of patients are at an advanced stage when diagnosed 3, which also leads to a 5-year survival rate of only 46% 4. With the progress of research, the management of ovarian cancer at present has transformed into a more precise and individualized application of cytoreductive surgery, platinum-based chemotherapy, and a combination of targeted therapies 5. The development of novel tumor prognostic and therapeutic biomarkers will help promote the continued development of precision medicine, which is the current trend in cancer research.

Pyroptosis, also known as inflammatory necrosis, is a type of programmed cell death. It is a special way of death induced in macrophages, neutrophils, and other phagocytes when the body is fighting against a pathogenic invasion 6, which is mainly divided into classical caspase-1 dependent pathways and non-classical caspase-4, 5, and 11 dependent pathways. Activated Caspase-1 or Caspase-4, 5, 11 can split Gasdermin D (GSDMD) into N- and C-terminals, where the N terminal (GSDMD-N) forms a transmembrane pore, on the one hand, allowing water molecules outside the cell to flow in, resulting in cell rupture. On the other hand, inflammatory factors such as IL-1β and IL-18 in cells are released, leading to a strong inflammatory response 6. However, in recent years, many researchers have found that this special programmed cell death can occur not only in immune cells, but can also be induced in a variety of cancer cells under the action of many chemotherapy drugs or molecules in a GSDMD 7 or GSDME 8-12, GSDMC 13 and GSDMB 14, 15 mediated manner. This shows great potential for inducing pyroptosis in tumor cells as a therapeutic method. The relationship between pyroptosis and cancer is more complicated. On the one hand, as a method of inducing cell death, pyroptosis of tumor cells can inhibit the occurrence and development of cancer. On the other, as a way of cell inflammatory death, pyroptosis can have a non-negligible impact on the tumor immune microenvironment, which in turn affects the occurrence and development of tumors. However, the impact of pyroptosis on the tumor immune microenvironment remains inconclusive. Even if the same type of cell undergoes pyroptosis, its anti-tumor or tumor-promoting effect depends on the relationship between cells undergoing pyroptosis, inflammasomes and anti-tumor immunity 6. Some studies suggest that pyroptosis can activate the tumor microenvironment to an immunostimulatory state, lead to tumor regression in a cytotoxic lymphocyte-dependent manner, and play an important role in anticancer immunity 16, 17. In addition, pyroptosis has also been found to act synergistically with immune checkpoint inhibitors to elicit protective immune responses 17. In contrast, studies have shown that chronic induction of pyroptosis may lead to chronic inflammation, resulting in a tumor-promoting microenvironment 18, 19. Elucidating the relevant mechanisms of pyroptosis in tumors will help to develop new anti-tumor therapies.

Long non-coding RNA (lncRNA) is a kind of non-coding transcript with more than 200 nucleotides. Although lncRNAs cannot be directly translated into proteins, they can affect gene expression by affecting DNA replication, transcriptional regulation, and post-transcriptional regulation, thus play an unignorable regulatory role in various life activities of cells 20. Previous studies have shown that in ovarian cancer, a variety of lncRNAs are dysregulated, which can play a role in cancer cell invasion, metastasis, and chemotherapy resistance, and may be used as cancer biomarkers 21. However, the current research on the role of lncRNA in tumor cell pyrolysis is still at an early stage, and the research on lncRNA in ovarian cancer cell pyroptosis is particularly limited.

**Figure 1:**
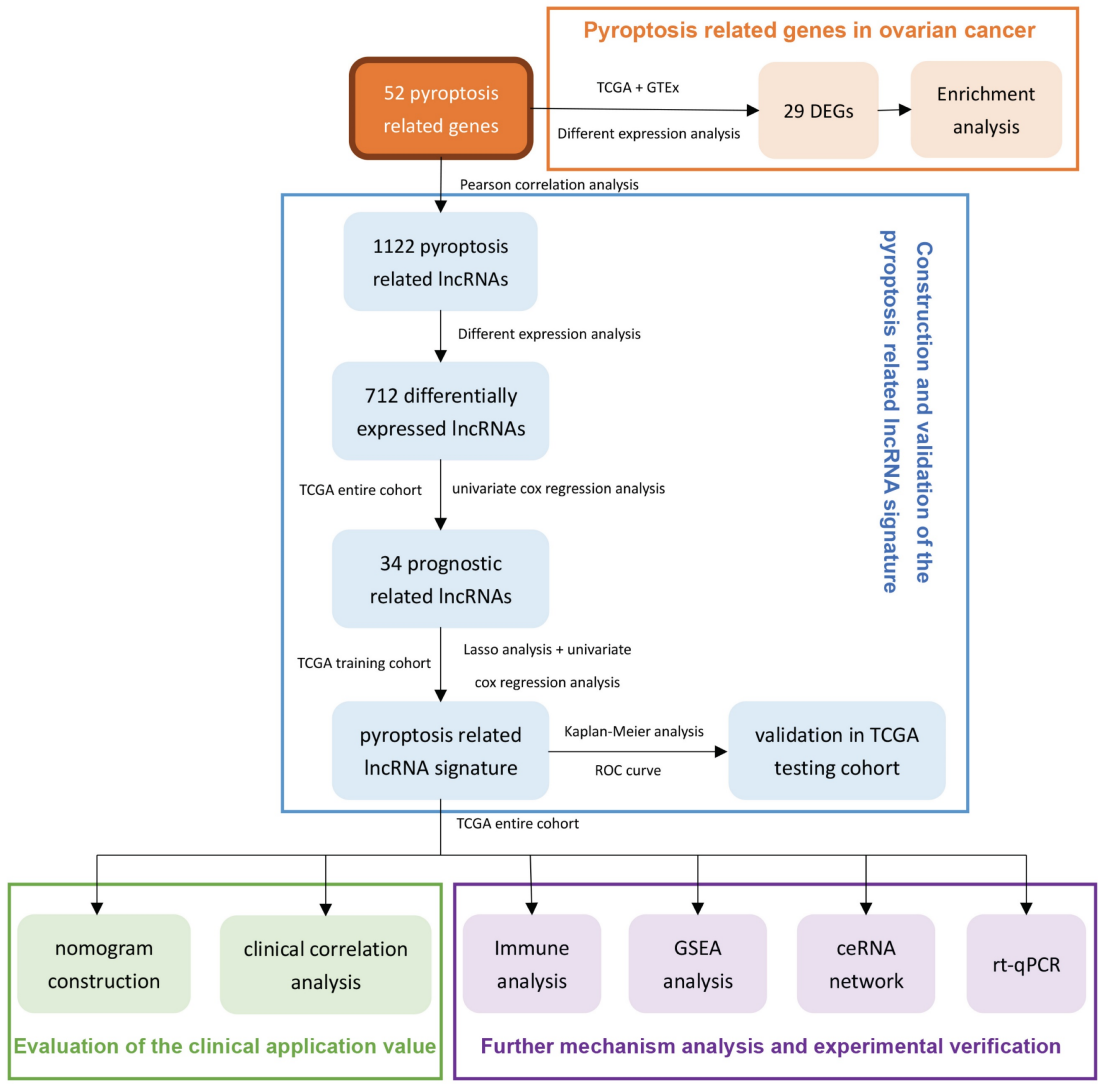
The flowchart of this research.

Therefore, we use the ovarian cancer data in TCGA to comprehensively analyze the lncRNAs related to pyroptosis in this research. After prognostic analysis, emerging prognostic markers and potential therapeutic targets related to pyroptosis were obtained, and a prognostic signature with clinical application value was constructed. In addition, we also explored the potential pathways of pyroptosis-related lncRNAs in ovarian cancer and the ceRNA network that interacts with them, providing a basis for elucidating the molecular mechanism of pyroptosis in ovarian cancer. In addition, real time quantitative PCR (rt-qPCR) was applied to verify the expression levels of two lncRNAs between three ovarian cancer cell lines and one normal ovarian cell lines. Figure 1 summarizes this process.

## 2. Materials and methods

### 2.1 Data collection

We downloaded the transcriptome expression data (RNA-seq, FPKM) of 427 patients with ovarian cancer from The Cancer Genome Atlas database (TCGA, https://portal.gdc.cancer.gov). Among them, 379 samples had complete Clinical information. We have obtained the gene expression data (RNAseq-TOIL RSEM expected counts) of 88 normal ovarian tissues in the Genotype-Tissue Expression (GTEX) database from UCSC Xena website (https://xenabrowser.net). The "normalizeBetweenArrays" package of R was used to normalize the data from TCGA and GTEX, and then merge the data for subsequent difference analysis. The RNAseq data included both mRNA and lncRNA transcriptome data and the Ensembl human genome browser GRCh38.p13 (http://asia.ensembl.org/index.html) was introduced to distinguish them.

### 2.2 Identification of dysregulated pyroptosis-related genes and lncRNAs between ovarian cancer and normal ovarian tissue

Fifty-two pyroptosis-related genes (PRGs) were obtained from existing studies 22-25 (Supplementary Table 1). We analyzed the expression correlation between lncRNA and pyroptosis genes based on the TCGA ovarian cancer transcriptome data. Taking the absolute value of Pearson's correlation coefficient≥0.30 and P-value<0.01 as the thresholds, we identified 1122 lncRNAs (Supplementary Table 2) that had significant expression correlation with pyroptosis genes. Using the "limma" R package, we obtained pyroptosis related genes and lncRNAs with significant expression differences between malignant and normal ovarian tissues according to a threshold of false discovery rate (FDR)<0.05 and |log2FoldChange|≥1.

### 2.3 Functional enrichment analysis of the dysregulated PRGs

An R package named "clusterProfiler" was applied to conduct Gene Ontology (GO) and Kyoto Encyclopedia of Genes and Genomes (KEGG) enrichment analysis on 29 PRGs. The enrichment results with P-value<0.05 and q-value<0.05 were considered to be statistically significant and visualized with the "ggplot" and "GOplot" R package packages.

### 2.4 Development and verification of a pyroptosis-related lncRNA risk signature

#### 2.4.1 Construction and verification of a pyroptosis-related lncRNA risk signature in the training and testing groups

First, the univariate Cox regression analysis was performed to identify pyroptosis-related lncRNAs that are significantly associated with the overall survival of ovarian cancer patients (P<0.05). Next, we grouped the ovarian cancer samples with complete clinical information in the TCGA into two groups. After deleting five duplicate samples of ovarian cancer patients, we separated the remaining 374 samples into a training group (n= 225) and a testing group (n=149) at a ratio of 6:4. In the training group, we used the "glmnet" R package to conduct LASSO analysis on 34 prognostic-related lncRNAs, and further screened and removed them based on the best penalty factor (λ). Finally, through stepwise multivariate Cox regression analysis, we conducted a final screening of these lncRNAs and established a risk signature consisting of 13 LncRNAs. The risk score for each patient was assessed according to the following calculation formula:



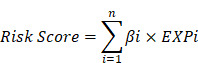



In the above formula, n indicates the number of lncRNAs in the signature, β indicates the coefficient of each lncRNA, and EXP indicates the detected expression level of each lncRNA. Patients in the training and testing cohorts were divided into high- and low-risk groups based on the median risk score. The overall survival rates of the high- and low-risk groups was compared by log-rank test of Kaplan-Meier analysis. The value of risk scores for predicting 1-, 3-, 5-, and 10-year survival in ovarian cancer patients was analyzed using time-dependent ROC curves by the "survivalROC" package.

#### 2.4.2 Prognostic value analysis of the signature

To assess whether risk score can be used as an independent risk factor for predicting prognosis in patients with ovarian cancer, we performed univariate and multivariate cox regression analyses using “Survival” and “SurvMiner” R packages for risk score, age, FIGO stage, grade of differentiation, tumor residue, and race in the training and testing groups. Using the "survivalROC" package, a time-dependent ROC curve was used to compare the predictive value of risk score against patient age, FIGO stage, pathological grade, and tumor residual for 1 -, 3 -, 5 -, and 10-year survival.

To better apply the risk score to the clinic, we used the "survival" and "regplot" packages to draw a nomogram in the overall cohort based on risk score, age, FIGO stage, differentiation grade, and tumor residual status. The calibration curve was used to assess the agreement between the predicted overall survival and the actual one. Using the "rms" and "rmda" packages, we also conducted decision curve analysis (DCA) to compare the effectiveness of nomogram and risk score and other individual clinicopathological indicators in predicting patient prognosis.

### 2.5 Analysis of the immune landscape

Using the "ESTIMATE" R package, we calculated the immune score and stromal score of each sample in TCGA-OV based on the expression profile data, and the sum of the two is the ESTIMATE total score. Finally, the tumor purity of each sample was assessed based on the above scores, that is, the higher the immune and stromal score, the lower the tumor purity 26. From the TIMER2.0 (http://timer.comp-genomics.org) website, we downloaded the infiltration scores of various immune cells in the samples from TCGA database calculated by seven platforms including TIMER, CIBERSORT, Cibersort-ABS, QUANTISEQ, MCPCOUNTER, XCELL and EPIC. In addition, we used single-sample gene set enrichment analysis (ssGSEA) to assess the different immune function scores of each sample in TCGA-OV 27.

### 2.6 GSEA for the high- and low-risk groups

We obtained Hallmark gene sets (hav7.4.symbols.gmt) and KEGG gene sets (c2.cp.kegg.v7.4.symbols.gmt) from the Molecular Signatures Database (MSigDB, https://www.gsea-msigdb.org/gsea/msigdb/index.jsp). Using GSEA software (version 4.0.3), the expression profiles of the high and low risk groups were compared with the two gene sets respectively, and the related HALLMARK and KEGG pathways related to the prognosis signature were analyzed. The number of random sample permutations was set to 1000. Using P<0.05 and FDR-q<0.25 as criteria, the pathways significantly associated with the high-risk group and the low-risk group were screened out, respectively.

### 2.7 Development of a ceRNA regulatory network for the lncRNAs in the signature

We first used Pearson's r≥0.2, P<0.05 as the threshold to identify PRGs that were significantly positively related to each lncRNA in the signature. In miRcode (http://www.mircode.org/) and starBase (version 2.0, https://starbase.sysu.edu.cn/) websites, miRNAs that interacted with lncRNA and PRGs were screened and compared to identify miRNAs that interacted with both lncRNA and PRGs. Finally, Cytoscape (Version 3.7.2) was introduced to visualize the ceRNA network. Among them, miRcode is a comprehensive website for deducing mircoRNA target sites in the complete GENCODE annotated transcriptome. It is mainly used to predict the interaction between lncRNA and microRNA, which contains 10419 lncRNAs 28. Starbase provides the most comprehensive miRNA-mRNA and miRNA-lncRNA interaction network supported by CLIP-Seq experiments, including about 10,000 pairs of ceRNA 29.

### 2.8 Cell culture and rt-qPCR

The ovarian cancer cell line (OVCAR3, SKOV3, and ES2) and normal ovarian cell line HOSEpiC were obtained from Shanghai Institute of Biochemistry and Cell Biology, Chinese Academy of Sciences (Shanghai, China). 10% fetal bovine serum (Biological Industries, Beit-Haemek, Israel) and 90% medium were prepared into complete medium for cell culture, among which OVCA3 and SKOV3 cell lines were cultured in RPMI 1640 medium (Biological Industries, Beit-Haemek, Israel), while ES2 and HOSEPIC cell lines were cultured in McCoy's 5A medium (Biological Industries, Beit-Haemek, Israel). The cells were incubated at 37 °C, 5% CO_2_ with condensate humidity. When the cells grew to 80-90% of the 6 cm dish, they were removed from the incubator and rinsed with PBS solution 1-2 times. Total RNA was extracted by TRIzol (Invitrogen, Carlsbad, CA, USA) and its concentration was detected by NanoDrop Lite spectrophotometer (Termo Scientifc). Total RNA was reverse transcribed using the PrimeScript™ RT Reagent Kit (TAKARA, RR047A, Shiga, Japan) for synthesis of cDNA. SYBR Green™ Premix Ex Taq™ II (TaKaRa, RR820A, Shiga, Japan) was used to amplify the samples on a real-time fluorescent quantitative PCR machine (Life Technologies, ABI7500fast). Each reaction well was provided with three different wells. The melting curve was used to determine the specificity of the primer. Using GAPDH as an internal reference, the relative expression level was calculated using the 2-△△Ct method. The primers of lncRNA and GAPDH are as follows: KRT7-AS-Forward: 5'-TCCAACGCCTATGTTCCAGTTC-3', KRT7-AS-Reverse: 5'-ACATTGTGCCACGGACATCTTG-3'; LINC01094-Forward: 5'-AATCCCAGTTGCTCTTCCAGTCATC-3', LINC01094-Reverse: 5'-CAGTGTTGTCCTCAGTTGCTCTCC-3'; GAPDH-Forward: 5'-GCACCGTCAAGGCTGAGAAC-3', GAPDH-Reverse: 5'-TGGTGAAGACGCCAGTGGA-3'.

### 2.9 Statistical analysis

All statistical analysis and visualization of results were conducted using R software (version 3.6.1), and p<0.05 or 0.01 was defined as statistically significant. Chi-square test was used to analyze count data, and Wilcox test was used to analyze measurement data. The Kaplan-Meier curve was used for survival analysis, and the log-rank method was applied to compare survival between the two groups. Cox regression model was used for univariate and multivariate analysis.

## 3. Results

### 3.1 Screening and differential analysis of lncRNA related to pyroptosis in ovarian cancer

**Figure 2:**
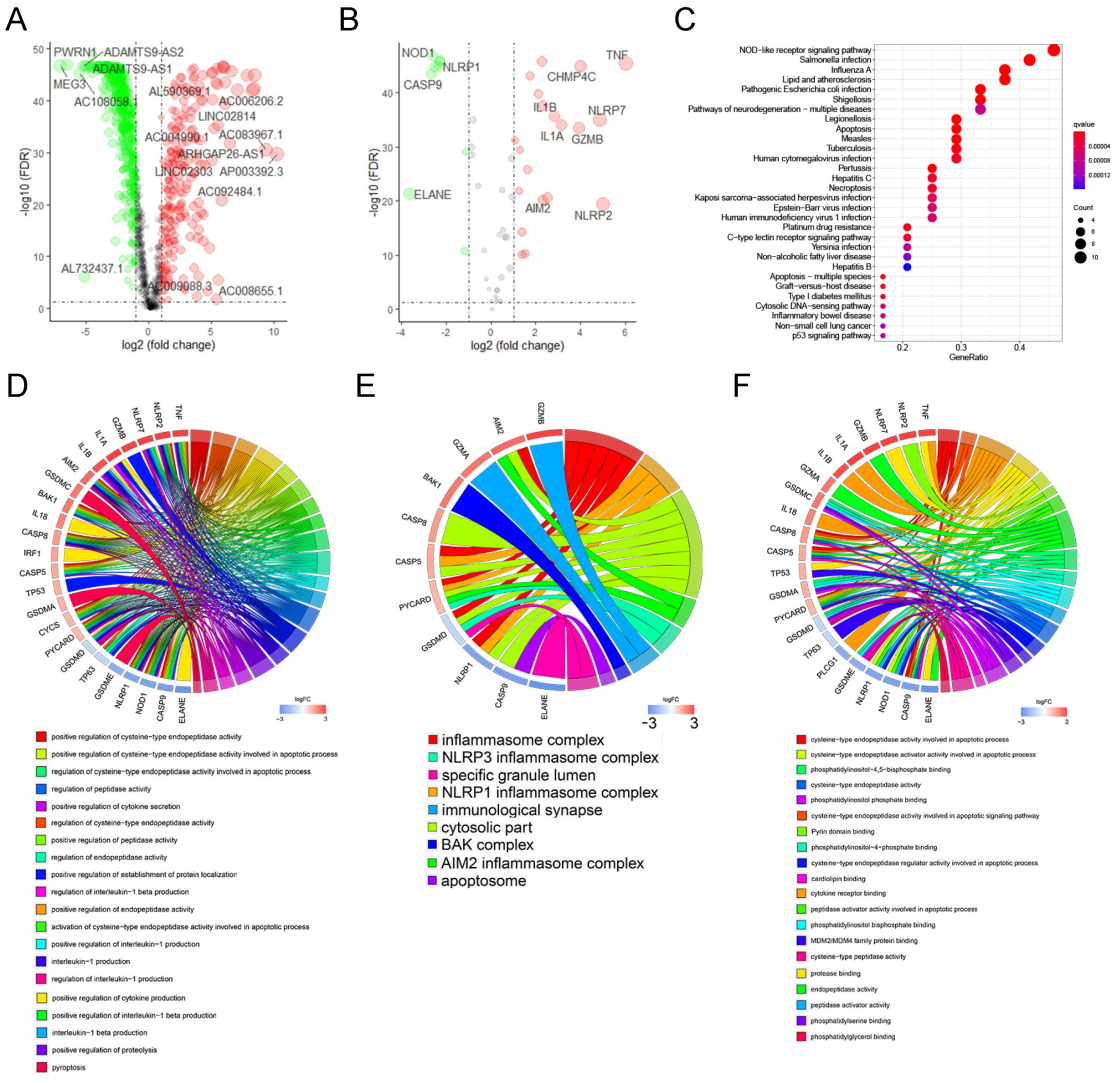
** Screening and functional enrichment analysis of pyroptosis-related genes and lncRNAs with dysregulated expression. (A)** The volcano graph of differential expression of pyroptosis-related lncRNAs between malignant and normal ovarian tissues, where the green dots represent the lnRNAs whose expression is significantly reduced in ovarian cancer (logFC≤ -1.0, FDR<0.05), and the red dots represent the lnRNAs that is significantly overexpressed in ovarian cancer (logFC≥1.0, FDR<0.05). In the figure, lnRNAs with │logFC│ ≥5.0 are marked. **(B)** The volcano graph of differential expression of PRGs between malignant and normal ovarian tissues, where the green and red dots represent the genes who are significantly down- or up- regulated in ovarian cancer (|logFC|≥ 1.0, FDR<0.05), respectively. In the figure, genes with │logFC│ ≥2.5 are marked. **(C)** The bubble chart of the KEGG enrichment analysis of 29 differentially expressed PRGs, where *P* value and the number of genes participate in the pathway is represented by the color and size of the bubbles, respectively. **(D-F)** Chord plot of biological process(D), cellular component(E), and molecular function(F) enrichment analysis results in GO enrichment analysis. Arranged according to the P value from small to large, the top 20 enrichment results and their related genes are displayed (of which there are only 9 meaningful results for molecular function). The genes are arranged according to their differentially expressed logFC value. lncRNAs: Long non-coding RNAs, PRGs: pyroptosis-related genes, FC: Fold Change, FDR: false discovery rate, KEGG: Kyoto Encyclopedia of Genes and Genomes, GO: Gene Ontology.

Using the transcriptome database of TCGA-OV, we identified 1122 lncRNAs that were significantly related to the expression of 52 PRGs with the absolute value of R≥0.30 and P<0.05 as the threshold. The correlation between these lncRNAs and PRGs is shown in Supplementary Table 2. Next, we performed differential expression analysis of these lncRNAs, and with the absolute value of logFC ≥ 1, FDR <0.05 as the threshold, we further identified 712 lncRNAs (Supplementary Table 3) that are significantly different expressed between normal and malignant ovarian tissues. Figure 2A shows the differential expression of these pyroptosis-related lncRNAs and annotates lncRNAs with an absolute value of logFC ≥ 5.0.

### 3.2 Differential analysis and pathway enrichment analysis of PRGs in ovarian cancer

Next, we analyzed the differential expression of 52 PRGs, using absolute value of logFC ≥ 1, FDR <0.05 as the threshold, and found 29 genes that are dysregulated between ovarian cancer and normal ovarian tissues. These include nine genes with significantly lower expression in ovarian cancer and 20 genes with higher expression in ovarian cancer (Supplementary Table 4). Figure 2B shows the differential expression of these PRGs and annotates genes with an absolute value of logFC ≥ 2.5.

We performed KEGG and GO enrichment analysis on 29 differentially expressed PRGs in ovarian cancer, with q-value≤0.05 as the threshold, and found that these genes were mainly enriched in apoptosis, NOD-like receptor signaling pathway, platinum drug resistance, and p53 signaling pathway. (Figure 2C). The GO enrichment results show that these genes are mainly enriched in the regulation of endopeptidase activity, pyroptosis and other biological processes (Figure 2D), and are mainly involved in the formation of cellular components such as inflammasome complex, cytosolic part, and immunological synapse (Figure 2E), and participate in cysteine-type endopeptidase activity involved in apoptotic process, cytokine receptor binding, protease binding and many other molecular functions (Figure 2F).

### 3.3 Development and verification of a pyroptosis-related lncRNA signature in ovarian cancer

In the overall sample of TCGA-OV, we selected 34 prognosis-related lncRNAs from 712 dysregulated pyroptosis-related lncRNAs by univariate-cox regression analysis (Supplementary Figure 1). Subsequently, we divided the overall sample of TCGA-OV into a training and a verification group (the number of samples were 225 and 149, respectively) according to a 6:4 ratio, which were then used for the establishment and verification of the signature, respectively.

#### 3.3.1 The development of the pyroptosis-related lncRNA signature

**Figure 3:**
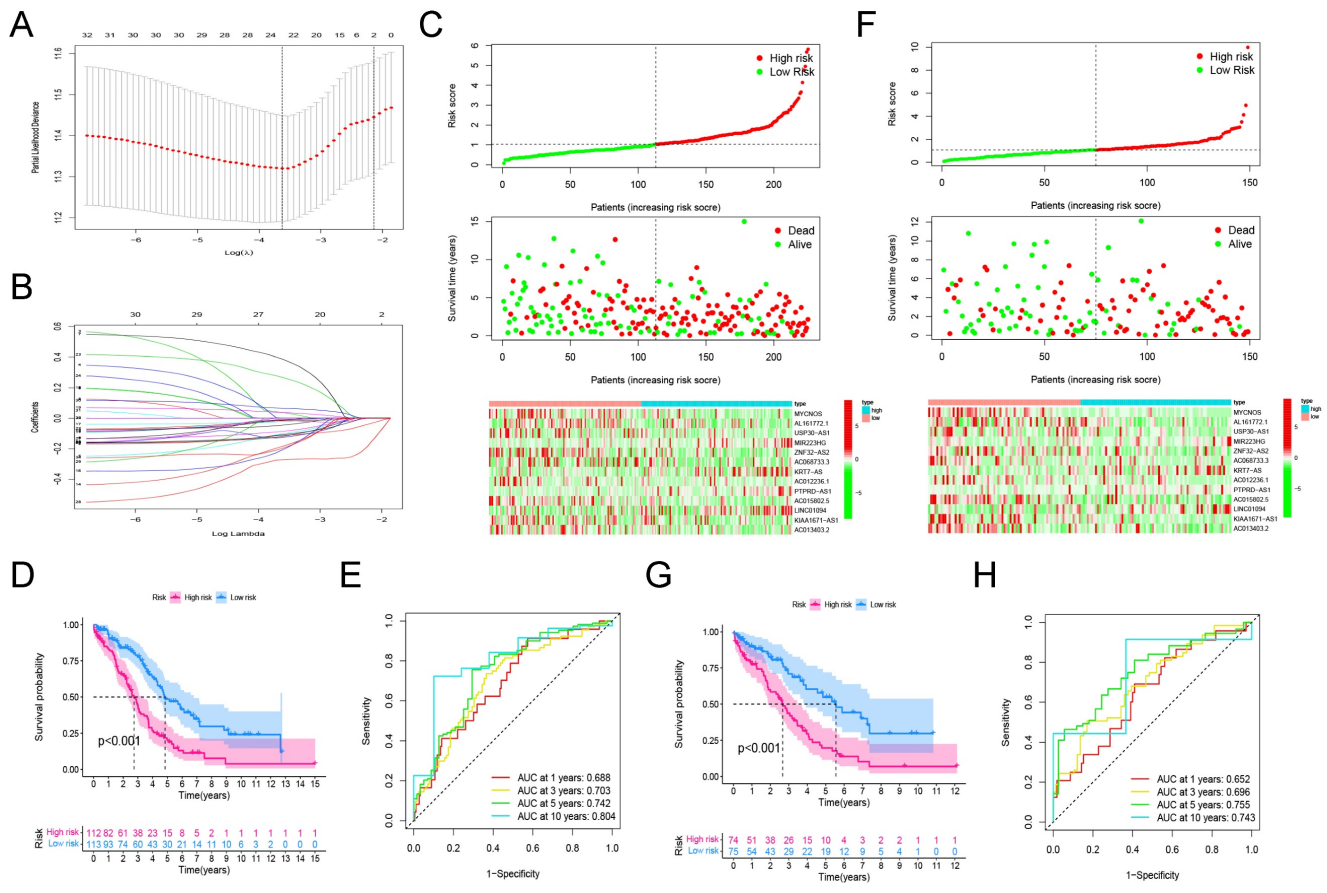
** The development and verification of a pyroptosis-related signature. (A)** The distribution of lambda values in LASSO analysis. **(B)** Coefficient distribution of LASSO regression analysis of 34 prognosis-related lncRNAs. **(C)** Plot of risk score (top), survival status (middle), and RNA expression heatmap (below) of patients in the training cohort. In the risk score graph, the risk scores of the low- and high- risk group are represented by the green and red curve, respectively. The green and red dots in the survival state diagram indicate the samples whose survival state is alive and dead, respectively. In the RNA expression heatmap, red and green indicates the up and down regulation of the lncRNA in the sample, respectively. **(D)** Kaplan-Meier survival analysis of high- and low-risk patients in the training cohort. **(E)** ROC curve for assessing the efficacy of our signature in predicting patients' 1-year, 3-year, 5-year, and 10-year survival rates in the training cohort. **(F)** Plot of risk score (top), survival status (middle), and RNA expression heatmap (below) of patients in the testing cohort. **(G)** Kaplan-Meier survival analysis of high- and low-risk patients in the testing cohort. **(H)** ROC curve for assessing the efficacy of our signature in predicting patients' 1-year, 3-year, 5-year, and 10-year survival rates in the testing cohort. LASSO: The Least Absolute Shrinkage and Selectionator operator, lncRNAs: Long non-coding RNAs, ROC: receiver operating characteristic curve.

Among the 225 samples in the training group, we conducted LASSO regression analysis on 34 prognostic-related lncRNAs, and selected 22 lncRNAs to be included in the subsequent stepwise multivariate cox regression analysis (Figure 3A, B). Finally, 13 lncRNAs were screened for the development of the signature. Table 1 shows the results of multivariate analysis, in which MYCNOS, AL161772.1, USP30-AS1, ZNF32-AS2, AC068733.3, AC012236.1, AC015802.5, KIAA1671-AS1 and AC013403.2 are the prognostic protective lncRNAs in ovarian cancer lncRNA (HR<1), while MIR223HG, KRT7-AS, PTPRD-AS1 and LINC01094 are the prognostic risk lncRNAs in ovarian cancer (HR>1). Using the expression of each lncRNA in the signature, multiplying by their respective coefficients, and finally adding up to calculate the risk score of each sample, that is, Risk score = (-0.142721*EXP_MYCNOS_) + (-0.176696*EXP_AL161772.1_) + (-0.1364156*EXP_USP30-AS1_) + (0.495127*EXP_MIR223HG_) + (-0.400991*EXP_ANF32-AS2_) + (-0.536123*EXP_AC068733.3_) + (0.052420*EXP_KRT7-AS_) + (-0.187234*EXP_AC012236.1_) + (0.369327*EXP_PTPRD-AS1_) + (-0.492214*EXP_AC015802.5_) + (0.099398*EXP_LINC01094_) + (-0.152476*EXP_KIAA1671-AS1_) + (-0.491793*EXP_AC013403.2_).

According to the above formula, we calculated the risk score of each patient in the training group and then defined them as high- and low-risk patients based on the median value (Figure 3C, top). There were more deaths in high-risk patients than in low-risk patients (Figure 3C, middle). Among the 13 LncRNAs in the signature, prognostic risk lncRNAs, MIR223HG, KRT7-AS, PTPRD-AS1 and LINC01094 were expressed higher in the high-risk patients than in the low-risk patients (Figure 3C, below). Prognostic protective lncRNAs (MYCNOS, AL161772.1, USP30-AS1, ZNF32-AS2, AC068733.3, AC012236.1, AC015802.5, KIAA1671-AS1 and AC013403.2) were on the contrary, expressed relatively highly in the low-risk patients (Figure 3C, below).

#### 3.3.2 Assessment of the efficacy of signature for predicting patient survival

To assess the efficacy of the signature in predicting ovarian cancer survival, Kaplan-Meier survival analysis was conducted in both high-risk and low-risk patients, which showed significantly poorer survival in the high-risk patients (P <0.001, Figure 3D). In addition, the result of the time-dependent ROC curve show that the signature has a good predictive efficacy on the patients' 1-year, 3-year, 5-year and 10-year survival rates (Figure 2E, the area under the curve (AUC) is 0.688, 0.703, 0.742 and 0.804, respectively).

#### 3.3.3 Verification of the prognostic signature in the testing group

In the validation cohort, we also separated patients into high- and low-risk groups according to risk scores (Figure 3F, Up). As shown in the middle and bottom part of Figure 3F, the number of patients whose survival outcome is death is more in the high-risk group than in the lower-risk group, and four prognostic risk genes have higher expression in the high-risk group, while the prognostic protection genes are the opposite. Similarly, the Kaplan-Meier curve of the validation cohort indicated that the prognosis of high-risk patients was significantly poorer than that of the low-risk patients (p<0.001, Figure 3G). As shown in Figure 3H, the risk signature can also accurately predict the survival rate in the validation cohort, whose 1-year, 3-year, 5-year, and 10-year area under the ROC curve are 0.652, 0.696, 0.755, and 0.743, respectively.

#### 3.3.4 Assessment of the clinical application value of prognostic signature

**Figure 4:**
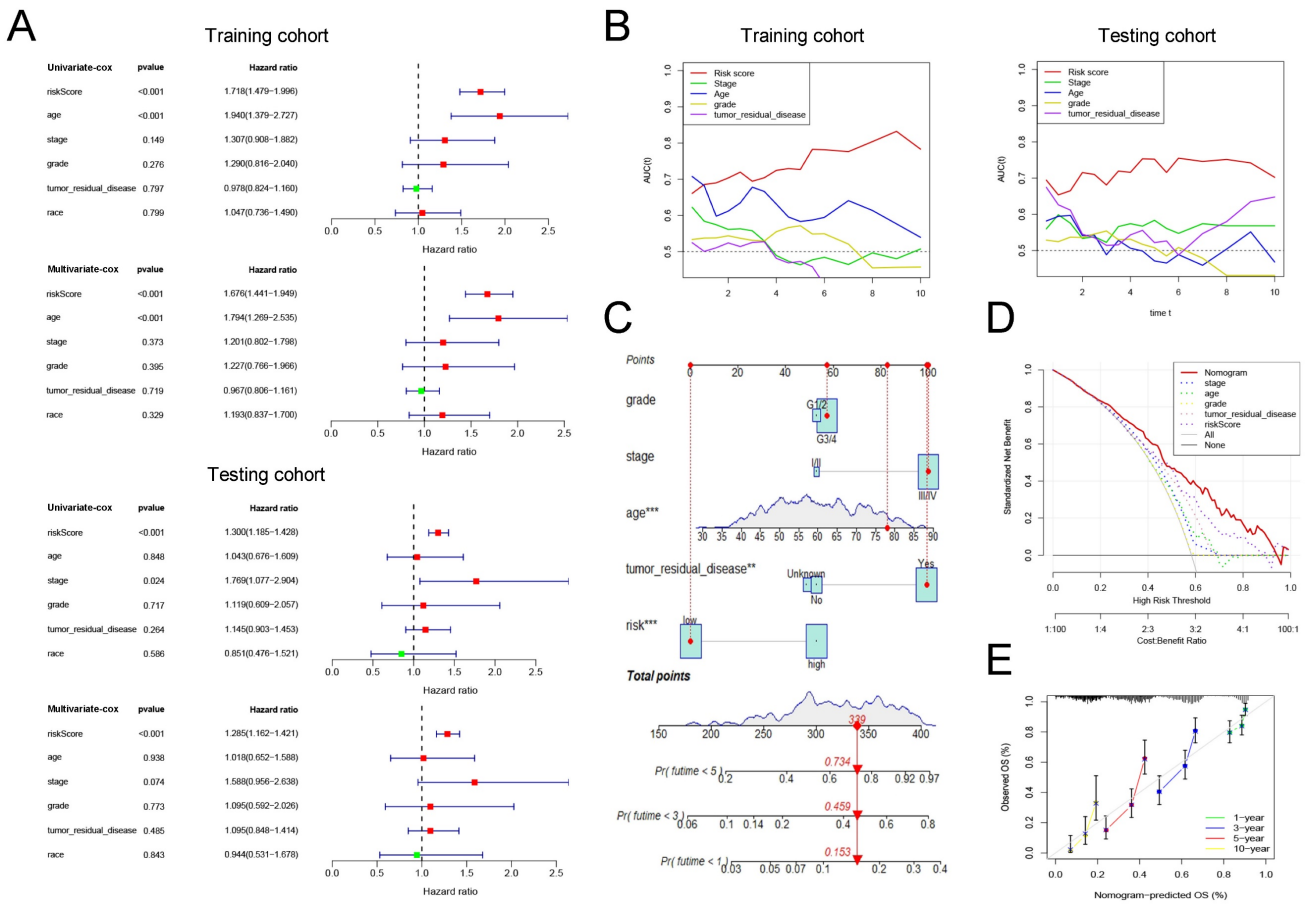
** The clinical application value of our pyroptosis-related signature. (A)** Univariate and multivariate Cox regression analysis of the risk score and other clinical factors containing age, FIGO stage, differentiation, tumor residual disease and race in the training and testing cohort. **(B)** Time-dependent ROC curves to compare the AUC values of risk score and other clinical factors including age, FIGO stage, differentiation, and tumor residual disease in time range from 1 year to 10 years in training and testing cohort. **(C)** A nomogram established in the entire cohort by combining risk score and other clinical factors containing age, FIGO stage, differentiation, and tumor residual disease. **(D)** DCA curve comparing the efficacy of nomogram, risk score, age, FIGO stage, differentiation, and residual tumor disease in predicting the survival rate of ovarian cancer patients in the entire cohort. **(E)** Calibration curve used to assess the agreement between nomogram-predicted survival and true survival of patients in the entire cohort. FIGO: Federation International of Gynecology and Obstetrics, AUC: Area Under Curve, DCA: Decision Curve Analysis.

Cox regression analysis was conducted on risk scores together with clinical indicators including age, FIGO stage, differentiation, tumor residual disease, and race in the training and testing group, respectively. The results of univariate cox regression analysis indicated that the risk score was significantly correlated with the prognosis of patients with ovarian cancer (Figure 4A, training cohort: P<0.001, HR=1.718; testing cohort: P<0.001, HR=1.300). Furthermore, the multivariate cox regression analysis indicated that the risk score may be an independent risk factor for ovarian cancer patients (Figure 4A, training cohort: P<0.001, HR=1.676; testing cohort: P<0.001, HR=1.285). Then, we drew time-dependent ROC curves in both the training group and the validation group to compare the AUC value of risk score and other clinical factors including age, FIGO stage, differentiation, tumor residual, and race in the time range of 1 to 10 years. The results show that, whether in the training or validation group, the risk score has better predictive power than other clinicopathological indicators (Figure 4B).

Finally, we combined the risk score and clinical indicators of age, FIGO stage, differentiation, and tumor residual to construct a nomogram to predict the survival of ovarian cancer patients in the overall cohort. As shown in Figure 4C, according to this nomogram, individual scores for age, FIGO stage, differentiation, tumor residual, and risk can be calculated according to the different conditions of each patient. Finally, the score obtained from the sum of the scores of each component can be used to predict the patient's 1-year, 3-year, and 5-year survival probability. Decision Curve Analysis (DCA) was carried out to evaluate the advantages of the nomogram in predicting the prognostic risk of ovarian cancer patients. As shown in Figure 4D, the effectiveness of nomogram was significantly better than risk score and other clinicopathological indicators. The efficacy of risk score alone was significantly higher than that of traditional clinicopathological indicators including age, FIGO stage, differentiation, and tumor residual. Finally, the results of the calibration curve showed that the 1 -, 3 -, 5 -, and 10-year survival rates estimated by the nomogram were close to the actual one, further indicating that this nomogram could predict the prognosis of ovarian cancer patients accurately (Figure 4E).

### 3.4 Robustness analysis of prognostic signature and its association with clinicopathological features

**Figure 5:**
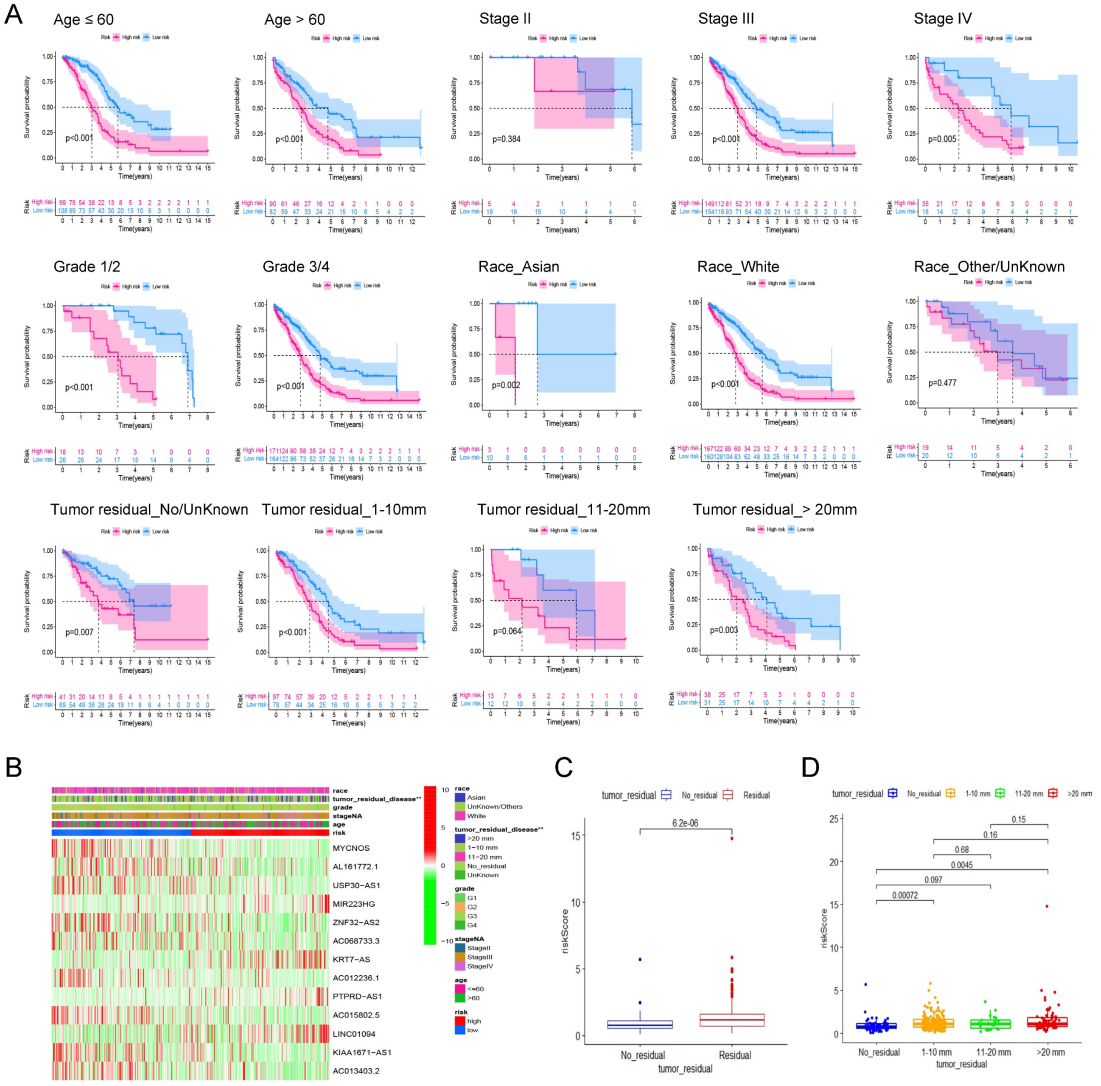
** The robustness analysis of our pyroptosis-related signature and its correlation with clinicopathological indicators of ovarian cancer patients. (A)** Robustness assessment of our signature. Kaplan-Meier survival analysis for high and low risk patients in the groups of younger than 60, older than 60, Stage II, Stage III, Stage IV, Grade 1 and 2, Grade 3 and 4, Asian race, White race, Other races or unknown, without tumor residual diseases or unknown, with a residual disease of 1-10 mm, 11-20 mm, and more than 20 mm, respectively. **(B)** The heat map shows the correlation between the expression profile of the lncRNA in the signature and the clinicopathological characteristics of patients. Different clinicopathological parameters and risk groups are displayed in different colors. The expression levels of the 13 lncRNAs are also indicated by color code bars, where red and green means up- and down-regulation, respectively. **(C)** Box plot of the difference in risk scores between patients without tumor residual diseases and those with tumor residual diseases.** (D)** Box plot of the difference in risk scores between patients without tumor residual diseases, with a tumor residual disease of 1-10 mm, 11-20 mm, and more than 20 mm, respectively.

To assess the robustness of the prognostic signature in clinical application, we first separated patients into different groups based on the clinical features including age (≤60, >60), FIGO Stage (Stage II, III, IV), Grade (Grade 1/2, 3/4), Race (Asian, White, Other/Unknown) and Residual tumor (No residual, 1-10 mm, 11-20 mm, >20 mm) and then performed kaplan-Meier survival analysis in each group based on risk scores. In addition to Stage I (P = 0.384, n = 23), Other/Unknown Race (P = 0.477, n = 39), tumor residual 11-10 mm (P = 0.064, n = 25) these three groups, the pyroptosis related signature can distinguish the overall survival of patients according to the risk scores (Figure 5A). Therefore, we believe that this prognostic signature can differentiate the prognosis of different patients independent of different clinical characteristics.

Then, heatmap was applied based on the risk score grouping and clinicopathological features of the patients. As shown in Figure 5B, there were significant differences in different size of residual tumor disease between the high and low risk groups, whereas no differences were observed between the other clinical characteristics (age, FIGO grade, stage, and race) that were included in the analysis. Thus, we visualized the difference in risk scores between patients with different tumor residual disease. The results showed that the risk score of patients with residual tumor was significantly higher than that of patients without residual tumor (Figure 5C, P = 6.2E-06). Further grouping analysis showed that the risk scores of patients with 1-10 mm tumor residual and >20 mm tumor residual were significantly higher than those without tumor residual (Figure. 5D, P = 0.00072 and 0.0045 respectively).

### 3.5 Correlation between signature and immune-infiltrating microenvironment of ovarian cancer patients

**Figure 6:**
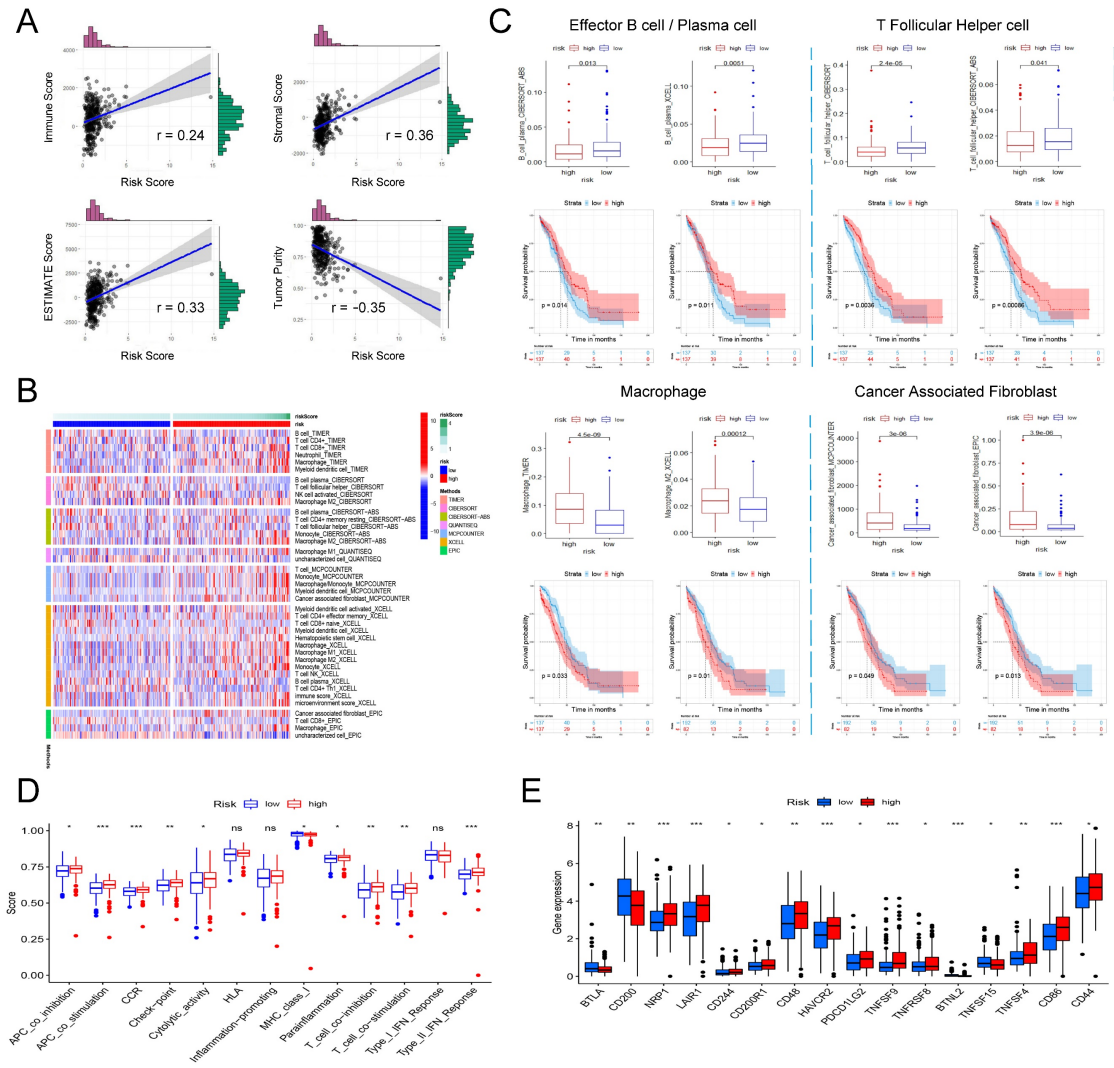
** The involvement of the signature in the immune microenvironment of patients with ovarian cancer. (A)** Scatter plot of the correlation between risk score and immune score, stromal score, ESTIMATE score as well as tumor purity. **(B)** Heatmap showing the differences in the infiltration of different immune cells between the high- and low-risk patients in seven calculation tools. The risk score, risk grouping and different calculation tools are all distinguished by different colors. Samples with high levels of immune cell infiltration are shown in red and vice versa in blue. **(C)** The difference in the infiltration of effector B cells, follicular helper T cells, macrophages and tumor-associated fibroblasts between the high and low risk groups, and the Kaplan-Meier curve comparing the effects of these immune cell infiltrations on the survival of ovarian cancer patients. **(D)** Differences in immune functions between high- and low-risk patients. **(E)** Differences in expression of immune checkpoints between high- and low-risk patients.

Based on genome-wide expression, we calculated Immune Score, Stromal Score, and tumor purity for each patient using the "ESTIMATE" R package. The sum of Immune Score and Stromal Score is ESTIMATE Score, which can be used to comprehensively evaluate the infiltration of immune cells and stromal cells in the microenvironment of tumor samples. As shown in Figure 6A, the risk score is significantly positively correlated with Immune Score (Pearson's r = 0.24, P = 7.55e-06), Stromal Score (Pearson's r = 0.36, P = 1.02e-11) and ESTIMATE Score (Pearson's r = 0.33, P = 8.83e-10), that is, patients with higher risk scores have higher immune cells and stromal cell infiltration. Correspondingly, high risk patient has a lower tumor purity (Figure 6A, Pearson's r = -0.35, P = 7.05e-11).

Next, we further explored the relation between the risk score and the infiltration of different immune cells evaluated by seven tools: TIMER, CIBERSORT, CIBERSORT-ABS, QUANTISEQ, MCPCOUNTER, XCELL and EPIC. Figure 6B shows all immune cells with significant differences in infiltration between the high and low risk groups (P <0.05). Based on the analysis of the prognostic information of the patient, we found that in ovarian cancer, patients with higher infiltration of effector B cells (also known as plasma cells) and follicular helper T cells had a better prognosis, and correspondingly, high risk patients had higher infiltration of effector B cells and follicular helper T cells (Figure 6C). In addition, we also found that in all seven platforms, the analysis showed that the high-risk patients had significantly higher macrophage infiltration, especially M2 type macrophages (Figure 6B). Further prognostic analysis results also indicated that patients with higher macrophage/M2 macrophage infiltration have a significantly poorer prognosis (Figure 6C). Finally, we also found that a higher risk score was significantly correlated with a richer tumor-associated fibroblast infiltration, and its higher infiltration was also significantly correlated with a shorter overall survival of the patient (Figure 6C).

Finally, we analyzed whether risk scores were associated with some immune-related functions and immune checkpoints in patients. ssGSEA was introduced to calculate the different immune function scores of each sample in TCGA-OV. The results are shown in Figure 6D. The higher risk score is significantly positively related to immune functions such as APC co inhibition, APC co stimulation, CCR, check point, cytolytic activity, parainflammation, T Cell co-stimulation, and Type II IFN response, while lower risk scores are significantly positively correlated with MHC class I. Further research on immune checkpoints found that the expression of some immune checkpoints such as BTLA, CD200, BTNL2, and TNFSF15 were significantly higher in the low-risk group, while NRP1, LAIR1, CD244, CD200R1, CD48, HAVCR2, PDCD1LG2, TNFSF8, TNFRSF8, TNFSF4, CD86 and CD44 and other immune checkpoints were significantly overexpressed in the low-risk group (Figure 6E).

### 3.6 GSEA enrichment analysis identifies signature-related pathways

**Figure 7:**
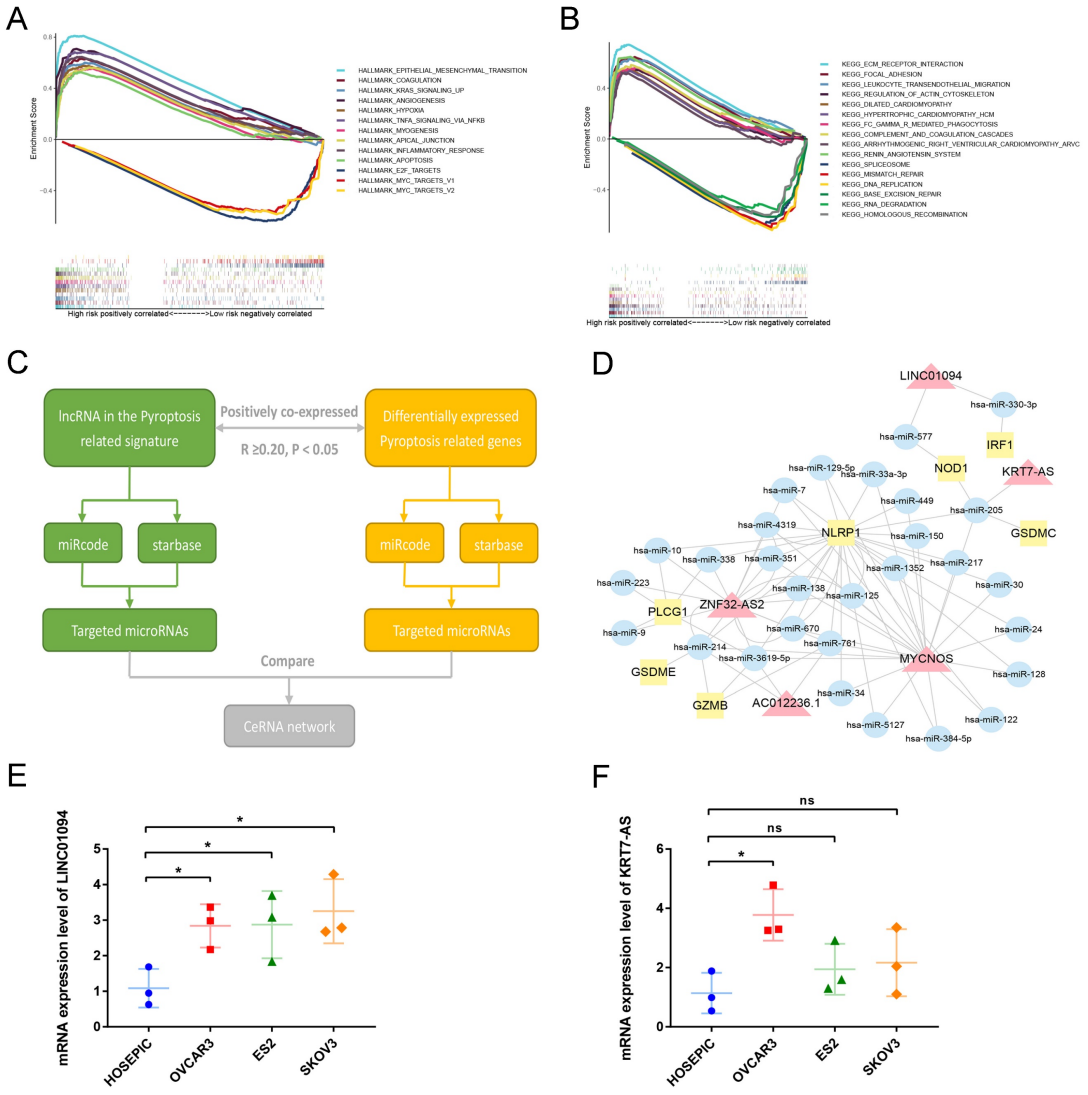
** Analysis of the mechanism of the signature. (A)** GSEA results show the top 10 significant enriched HALLMARK pathways positively correlated with the high-risk ovarian cancer patients and 3 significant enriched pathways negatively correlated with the low-risk ovarian cancer patients. **(B)** GSEA results show the top 10 significant enriched KEGG pathways positively correlated with the high-risk ovarian cancer patients and 3 significant enriched pathways negatively correlated with the low-risk ovarian cancer patients. **(C)** The process of constructing ceRNA regulatory network related to pyrolysis in ovarian cancer. **(D)** The ceRNA regulatory network showing the interactions between pyroptosis-related LncRNAs in our signature and PRGs. The red triangle represents lncRNA, the yellow square represents mRNA, and the blue circle represents microRNA. **(E, F)** The mRNA expression levels of LINC01094 and KRT7-AS between normal ovarian cell line HOSEPIC and ovarian cancer cell lines (OVCAR3, ES2 and SKOV3) by rt-qPCR. GSEA: Gene set enrichment analysis, KEGG: Kyoto Encyclopedia of Genes and Genomes, ceRNA: competing endogenous RNA, lncRNA: Long non-coding RNA. * P<0.05.

To analyze the downstream pathways that the signature may participate in, we used GSEA to compare the genetic characteristics of the high- and low-risk groups with the various pathways of HALLMARK and KEGG. With normalized P-value<0.05 and FDR q-value<0.25 as thresholds, we screened the pathways related to the two groups, respectively. Sorted by Enrichment Score, Figure 7A shows the top 10 pathways positively associated with the high-risk group and the three pathways negatively associated with the low-risk group in the HALLMARK enrichment analysis. The results show that ovarian cancer in the high-risk group may be involved in epithelial-mesenchymal transition, KRAS signaling pathway, TNF NFKB signaling pathway, and angiogenesis, while the low-risk group is negatively associated with E2F and MYC pathways. Similarly, KEGG enrichment show that ovarian cancer in the high-risk group may be involved in ECM receptor interaction, focal adhesion, leukocyte trans-endothelial migration, and regulation of actin cytoskeleton, while the low-risk group is negatively associated with mismatch repair and DNA replication (Figure 7B).

### 3.7 Construction of ceRNA regulatory network of lncRNA in the pyroptosis-related prognostic signature

To further analyze the possible targets of lncRNAs in the pyroptosis-related signature and related regulatory axes in ovarian cancer, we combined the RNASeq transcriptome expression data of TCGA-OV with miRcode and starBase databases to predict and construct a competing endogenous RNA (ceRNA) regulatory network. The ceRNA regulatory network has been proven by many studies to play an important role in a variety of tumors. The theoretical basis is that certain mRNA and lncRNA in the cell have the same miRNA response element, and a competitive relationship can be formed between the two. Since miRNA has a negative regulatory effect on the expression of its target genes, the expression of a pair of competitive mRNA and lncRNA with the same miRNA response element is positively correlated. The process of constructing a ceRNA regulatory network related to pyroptosis in ovarian cancer is shown in Figure 7C. First, among the differentially expressed PRGs in ovarian cancer, we screened mRNAs that are significantly positively correlated with lncRNA in the prognostic signature (R≥0.20, P<0.05). Then use the miRcode and starbase database to predict the target miRNA, and finally screen out the miRNA that has an interaction relationship with both lncRNA and mRNA to construct the network. As shown in Figure 7D, among the 13 lncRNAs in the prognostic signature, 5 were found to have the same target miRNAs with their positively correlated PRGs, namely, LINC01094, KRT7-AS, MYCNOS, ZNF32-AS2 and AC012236.1. Through miRNA, these lncRNAs are connected with PRGs such as IRF1, NOD1, GSDMC, NLRP1, PLCG1, GSDME and GZMB to construct a pyroptosis related ceRNA regulatory network in ovarian cancer.

Next, we conducted preliminary rt-qPCR verification on two poor prognosis-related lncRNAs in this ceRNA network. The results indicated that compared with the normal ovarian cell line HOSEPIC, the expression of LINC01094 in the three ovarian cancer cell lines was significantly upregulated (Figure 7E, P<0.05). The expression of KRT7-AS only in the OVCAR3 cell line was significantly higher than that in HOSEPIC (Figure 7F, P<0.05), while the expression in ES2 and SKOV3 cell lines was not significantly different from HOSEPIC (Figure 7F, P>0.05). Using LnCAR database, we found that KRT7-AS was significantly overexpressed in ovarian cancer tissues compared with normal.

## 4. Discussion

Pyroptosis was originally found in pathogen-infected mouse macrophages and monocytes 30, 31, but it was only recently discovered that it can also occur in healthy cells and cancer cells. As numerous studies have found that it can be induced in tumor cells, its potential role in anti-cancer therapy has received extensive attention. In ovarian cancer, studies have found that Osthole 32, Nobiletin 33, 2‐(α-naphthoyl) ethyl‐trimethylammonium iodide (α -NETA) 7 and lncRNA GAS5 34 has the effect of inducing pyroptosis, while the inhibition of miRNA-15a 35 and lncRNA HOTTIP 36 have the function of activating the related pathways of pyroptosis. However, these current studies are still only the tip of the iceberg. A more comprehensive study of the mechanism of pyroptosis in ovarian cancer will help to develop novel and effective anti-cancer therapies, which is the general trend of current research.

In this work, we identified 29 PRGs with dysregulated expression level between ovarian cancer and normal ovarian tissues. In the gasdermin family, GSDMA (logFC=1.38557, FDR=5.23e-15) and GSDMC (LogFC=2.298323, FDR=2.06e-38) was significantly overexpressed in ovarian cancer, while the expression of GSDMD (logFC=-1.10275, FDR=8.25e-30) and GSDME (logFC=-2.29572, FDR=1.54e- 46) was significantly lower in ovarian cancer (Supplementary Table 3). At present, studies have shown that GSDMD 7, 33 and GSDME 32, 33 are the key targets that cause pyroptosis in ovarian cancer cells, while GSDMA and GSDMC have not been proven to be involved in the pyroptosis of ovarian cancer cells, which is consistent with our analysis results. Through KEGG enrichment analysis of 29 differentially expressed PRGs, we found that they were enriched in Apoptosis, NOD-like receptor signaling pathway, Platinum drug resistance, p53 signaling pathway and other pathways, while Biological Process enrichment analysis found them mainly involved in regulation of endopeptidase activity and pyroptosis. These results indicate that the PRGs we screened are indeed involved in the role of pyroptosis and are also related to tumor-related pathways such as NOD-like receptor signaling pathway, p53 signaling pathway, and platinum drug resistance.

Next, we screened the lncRNAs related to pyroptosis and performed prognostic analysis on the differentially expressed ones. After further screening by LASSO and multivariate cox stepwise regression analysis, 13 lncRNAs were included in the final signature construction, including four ovarian cancer prognostic risk lncRNAs (MIR223HG, KRT7-AS, PTPRD-AS1 and LINC01094), and 9 prognostic protection lncRNAs (MYCNOS, AL161772.1, USP30-AS1, ZNF32-AS2, AC068733.3, AC012236.1, AC015802.5, KIAA1671-AS1 and AC013403.2). MIR223HG is also known as LINC223. Studies have found that its expression in acute myeloid leukemia is significantly down-regulated and has the function of inhibiting cell cycle progression and promoting the transformation of acute myeloid leukemia cells into monocytes 37. KRT7-AS is an antisense RNA of the protein coding gene KRT7. Previous researches have proved that it can promote the progression of colorectal cancer 38, breast cancer 39 and gastric cancer 40. LINC01094 has been shown by many studies to promote the progression of various cancers by interacting with various miRNAs, including ovarian cancer 41, breast cancer 42, pancreatic cancer 43, renal clear cell tumor 44, glioma, etc. 45. MYCNOS, as an antisense RNA of coding gene MYCN, can play a role in the regulation of malignant tumors such as ovarian cancer 46, nephroblastoma 47, glioblastoma 48 and liver cancer 49 by regulating MYCN or other encoding genes. Similarly, USP30-AS1 is an antisense RNA of USP30, and its role in cancer regulation can depend on USP30 or other encoding genes. In acute myeloid leukemia, it can inhibit tumor by regulating the expression of USP30 50. Whereas in cervical cancer 51 and glioblastoma 52, USP30-AS1 has been shown to be related to cancer progression. These lncRNAs have been shown to be involved in the pathogenesis of many cancers in previous studies, but our research has discovered for the first time their potential role in pyroptosis of ovarian cancer cells. In addition, the research on other lncRNAs so far is very limited. Our study discovered for the first time their potential role in cancer, especially ovarian cancer related to pyroptosis.

Tumor microenvironment (TME) is a complex environment for the survival and development of tumor cells. It is mainly formed of surrounding immune cells, inflammatory cells, fibroblasts, stromal cells and microvessels, which play a role in assisting the occurrence, growth and metastasis of tumor. Our analysis found that the risk score of TCGA ovarian cancer samples was significantly positively associated with immune score and Stromal score, while significantly negatively correlated with tumor purity, indicating that the prognostic signature was involved in the tumor microenvironment of ovarian cancer. As a kind of inflammatory cell death, pyroptosis is the main mechanism of body defense. Cells will release a large amount of IL-18 and IL-1β when pyroptosis occurs, so it is essential for bridging innate immunity and adaptive immunity 53. Existing studies have shown that both the inflammatory cytokines released by pyroptosis and gasdermin family members have a regulatory effect on immune cells 54. However, the correlation between pyroptosis and immune infiltration in ovarian cancer remains unclear. By comprehensive analysis of the results of TIMER, CIBERSORT, XCELL and other platforms, we found that plasma cells and follicular helper T cells had higher infiltration in low-risk ovarian cancer patients, while macrophages and tumor-associated fibroblasts had higher infiltration in high-risk ovarian cancer patients. Prognostic analysis also showed that patients with higher plasma cells and follicular helper T cells infiltration had significantly better outcomes, while patients with higher infiltration of macrophages and tumor-associated fibroblasts had poorer outcomes. Follicular helper T cells are a type of CD4^+^T cells, which belong to anti-tumor immune cells 55, while the role of B cells in tumor immunity is still controversial, but in ovarian cancer, most studies have suggested that tumor infiltration of B cells is related to the improvement of prognosis of ovarian cancer patients 56. M1-type macrophages have long been considered to have good tumor antagonism, while M2-type macrophages are considered to be the main source of Myeloid-derived suppressor cells, which can not only inhibit immune surveillance of tumor cells, but also promote angiogenesis and matrix remodeling 57. Cancer-associated fibroblasts (CAFs) are an important part of the tumor microenvironment of ovarian cancer. It is transformed from resting fibroblasts under the interaction of mesenchymal stem cells and tumor cells and have the ability to promote tumor growth and invasion by secreting a variety of cytokines 56. Combining the above existing studies, high-risk patients are correlated with higher infiltration of tumor immunosuppressive cells such as M2 macrophages and CAFs, while low-risk patients are associated with higher infiltration of anti-tumor immune cells such as T cells and plasma cells. This indicates that lncRNAs in the pyroptosis related signature are related to tumor immune escape and has potential application value in tumor immunotherapy of ovarian cancer.

In addition, existing studies have shown that pyroptosis-related molecules are related to a variety of immunomodulatory effects, and the inflammatory factors IL1β 58 and IL18 59 released in the process can induce the differentiation of naive T cells and promote their proliferation. In addition, another inflammatory factor, HMGB1, can promote the migration of dendritic cells and induce the secretion of tumor necrosis factor by macrophages 60. GSDMB and GSDMC can be activated or upregulated by a variety of immune cells, leading to the occurrence of tumor cell pyroptosis 61, 62. The expression of GSDMD in cytotoxic T cells is positively correlated with its ability to eliminate tumor cells 63. These findings suggest that pyroptosis and its related molecules play a significant role in the anti-tumor effect of immune cells. Therefore, it is not surprising that our study revealed that the pyroptosis related signature is associated with a variety of immune functions, such as the activation and inhibition of antigen-presenting cells (APC), chemokine receptor (CCR), immune checkpoints, and activation and inhibition of T cells. A previous study found that GSDMA expression may increase the sensitivity of breast cancer cells against PD-L1 immunotherapy 64. We found that the expression of multiple immune checkpoints such as NRP1, LAIR1, HAVCR2, TNFRSF8, and CD86 was significantly upregulated in the high-risk group. On the one hand, it suggests that our pyroptosis-related lncRNAs signature may provide a good reference for immune checkpoint blocking therapy in patients with ovarian cancer. On the other hand, further analysis of the influence of these pyroptosis related lncRNAs on immune checkpoint suppression therapy is also a promising direction.

The ceRNA hypothesis was first proposed by Leonardo Salmena in 2011. It posits that competitive endogenous RNA (ceRNA) existing in cells can competitively bind to the same miRNAs through the same microRNA response elements (MRE), thus regulating each other's expression levels 65. Currently, studies on the regulatory mechanism of miRNA have been relatively mature. By binding with MRE on the transcript of the encoding gene, namely mRNA, miRNA leads to degradation of mRNA or inhibits its translation, thus negatively regulating gene expression levels. According to Leonardo Salmena's hypothesis, some other RNA molecules in cells, such as lncRNA, also have MRE, and can indirectly positively regulate the expression levels of the mRNA with the same MRE by competitively binding to their target miRNA. According to this hypothesis, we used the two microRNA target databases of miRcode and starbase, as well as the mRNA and lncRNA expression data in TCGA-OV, to construct a miRNA-centered ceRNA regulatory network. Thus, we discovered many unproven pyroptosis related regulatory axes in ovarian cancer, such as KRT7-AS—has-miR-205—GSDMC, LINC01094—has-miR-330-3p—IRF1, ZNF32-AS2—has-miR-214—GSDME, and MYCNOS—has-miR-150—NLRP1. Our rt-qPCR experimental results preliminarily prove that the expression of LNC01094 and KRT7-AS in ovarian cancer cell lines was higher than that in normal ovarian cell lines. These results provide an important basis for further studies on the mechanism of lncRNA in pyroptosis of ovarian cancer cells. In addition, through GSEA, we discovered that the samples in high-risk group had the highest correlation (Enrichment score) with epithelial mesenchymal transformation, ECM receptor interaction, and Focal adhesion. These results indicate that these pyroptosis-associated lncRNAs are likely to be involved in the metastasis of ovarian cancer cells, providing a direction for subsequent experimental studies.

## 5. Conclusion

In this study, 34 pyroptosis related lncRNAs that have an impact on the prognosis of ovarian cancer patients were identified through comprehensive analysis of TCGA database. The prognostic signature constructed using 13 pyroptosis related lncRNAs can not only accurately predict the survival of ovarian cancer patients but also is related to the high infiltration of macrophages and tumor-associated fibroblasts in ovarian cancer. In further analysis of the mechanism, we found that the signature may be related to the metastasis of ovarian cancer, and a pyroptosis related ceRNA network provides a basis for subsequent experimental studies. LNC01094 and KRT7-AS were observed to be overexpressed in ovarian cancer cell lines.

## Supplementary Material

Supplementary figure and table legends.

Supplementary tables.

## Figures and Tables

**Table 1:** Results of the 13 key LncRNAs in the stepwise multivariate Cox regression analysis

LncRNAs	coefficient	HR	HR.95L	HR.95H	*P*-value
MYCNOS	-0.142721	0.866996	0.753592	0.997465	0.045993
AL161772.1	-0.176696	0.838034	0.659009	1.065692	0.149566
USP30-AS1	-0.1364156	0.87248	0.785421	0.969188	0.010975
MIR223HG	0.495127	1.640707	1.09449	2.459518	0.016526
ZNF32-AS2	-0.400991	0.669656	0.397444	1.128307	0.131952
AC068733.3	-0.536123	0.585012	0.283245	1.20828	0.147418
KRT7-AS	0.05242	1.053818	1.012343	1.096993	0.010505
AC012236.1	-0.187234	0.829249	0.686395	1.001835	0.052263
PTPRD-AS1	0.369327	1.446761	1.08157	1.935259	0.012837
AC015802.5	-0.492214	0.611271	0.372932	1.001934	0.050902
LINC01094	0.099398	1.104506	0.990417	1.231737	0.07396
KIAA1671-AS1	-0.152476	0.85858	0.7537	0.978053	0.021802
AC013403.2	-0.491793	0.611529	0.35251	1.060869	0.080164

## Abbreviations

PRGs: pyroptosis-related genes
lncRNAs: Long non-coding RNAs
ceRNA: competing endogenous RNA
TME: Tumor microenviroment
CAFs: Cancer-associated fibroblasts
MRE: microRNA response elements
APC: antigen-presenting cells
CCR: chemokine receptor
TCGA: The Cancer Genome Atlas database
GTEX: Genotype-Tissue Expression
ssGSEA: single-sample gene set enrichment analysis
GSEA: Gene set enrichment analysis
KEGG: Kyoto Encyclopedia of Genes and Genomes
GO: Gene Ontology
FC: Fold Change
FDR: false discovery rate
LASSO: The Least Absolute Shrinkage and Selectionator operator
ROC: receiver operating characteristic curve
AUC: Area Under Curve
DCA: Decision Curve Analysis
FIGO: Federation International of Gynecology and Obstetrics
rt-qPCR: real time quantitative PCR
