# Supplementary material for: Identification of a pyroptosis-related long non-coding RNA Signature for prognosis and its related ceRNA regulatory network of ovarian cancer
Source: J Cancer. 2023 Sep 25;14(16):3151–68. doi: 10.7150/jca.88485 (PMC10583579; doi:10.7150/jca.88485)

Supplementary Material

Supplementary Figure 1. The forest map of 34 pyroptosis-related lncRNAs that is considered to be significantly correlated with the overall survival of patients with ovarian cancer after univariate-cox regression analysis.

Supplementary Table 1. The 52 pyroptosis-related genes included in our research.

Supplementary Table 2. The 1122 lncRNAs that have significant expression correlation with pyroptosis-related genes.

Supplementary Table 3. The 712 lncRNAs that are significantly different expressed between normal and malignant ovarian tissues.

Supplementary Table 4. The 29 pyroptosis-related genes that are dysregulated between ovarian cancer and normal ovarian tissues.

	pvalue	Hazard ratio
AC104825.1	0.041	0.848(0.723−0.994)
MYCNOS	<0.001	0.783(0.681−0.900)
AC087286.2	0.007	2.433(1.279−4.625)
AC138904.1	0.032	0.783(0.626−0.979)
AL161772.1	0.021	0.766(0.611−0.960)
USP30−AS1	0.041	0.905(0.823−0.996)
MIR223HG	0.001	1.888(1.292−2.758)
AL450384.2	0.047	0.822(0.678−0.998)
RPP38−DT	0.037	0.670(0.461−0.975)
AC134407.1	0.018	0.617(0.414−0.920)
AL355488.1	0.009	0.863(0.772−0.964)
AC092803.1	0.006	0.835(0.734−0.949)
SEMA3F−AS1	0.030	0.779(0.622−0.976)
ZNF32−AS2	0.018	0.584(0.374−0.912)
AL118511.1	0.024	0.725(0.548−0.958)
AC068733.3	0.032	0.486(0.252−0.939)
AC109322.1	0.024	0.922(0.859−0.990)
AC244197.2	0.048	0.854(0.730−0.998)
KRT7−AS	0.017	1.044(1.008−1.082)
AC012236.1	0.020	0.805(0.671−0.967)
AC004540.1	0.028	0.955(0.917−0.995)
DLGAP1−AS2	0.021	0.917(0.852−0.987)
PTPRD−AS1	0.006	1.450(1.115−1.885)
AP003419.3	0.022	0.707(0.526−0.951)
POLH−AS1	0.029	0.563(0.336−0.943)
LINC01993	0.032	0.627(0.409−0.960)
AC027348.1	0.004	0.783(0.664−0.923)
AC015802.5	<0.001	0.416(0.254−0.681)
LINC00702	0.043	1.327(1.009−1.744)
LINC01094	0.001	1.169(1.063−1.286)
AC109587.1	0.032	0.844(0.723−0.985)
LINC00893	0.045	0.969(0.940−0.999)
KIAA1671−AS1	0.011	0.860(0.766−0.966)
AC013403.2	0.023	0.552(0.331−0.923)

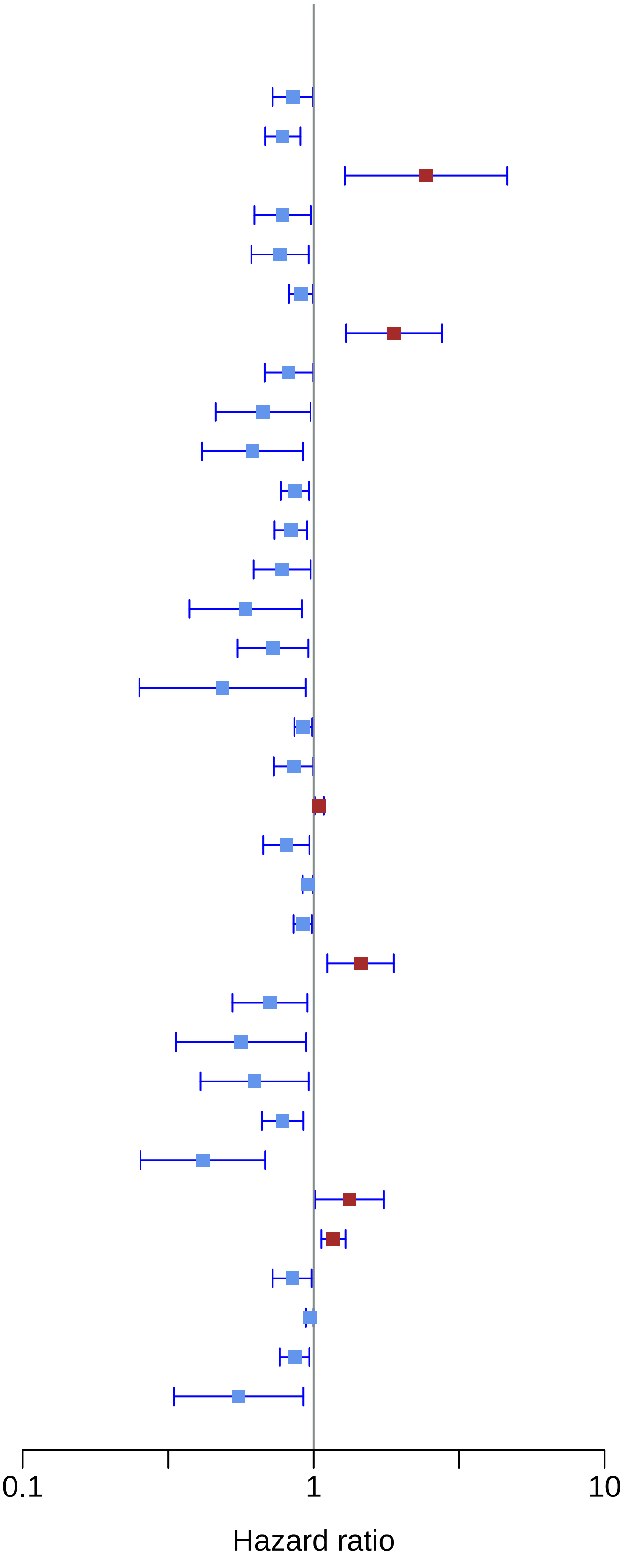

Supplement: Supplementary file 1 — Supplementary figure and table legends. [file jcav14p3151s1.pdf]
